# Diagnostic Value of MicroRNAs for Urologic Cancers: A Systematic Review and Meta-Analysis

**DOI:** 10.1097/MD.0000000000001272

**Published:** 2015-09-18

**Authors:** Hui Ouyang, Yanzhao Zhou, Li Zhang, Guanxin Shen

**Affiliations:** From Department of Immunology, Tongji Medical College, Huazhong University of Science and Technology, Wuhan, China (HO, LZ, GS); and Laboratory of Cardiovascular Immunology, Institute of Cardiology, Union Hospital, Huazhong University of Science and Technology, Wuhan, China (YZ).

## Abstract

MicroRNAs (miRNAs), particularly those extracted from the blood or tissues, have become the focus of urologic cancers research. However, the literature reviews on the accuracy of miRNA detection in urologic cancers have been inconsistent, leading us to perform this meta-analysis.

Eligible studies were searched in PubMed and other databases. To calculate the pooled detection accuracy estimates, we used a bivariate random-effects meta-analysis model.

According to the exclusion and inclusion criteria, 41 studies were included. Overall, the results showed sensitivity of 0.77 (95% CI: 0.74–0.80) and specificity of 0.76 (95% CI: 0.72–0.79), with an area under the SROC curve (AUC) of 0.83 (95% CI: 0.80–0.86). In addition, further subgroup analyses were also conducted. Firstly, the multiple miRNAs subgroup has significantly better diagnostic specificity than single miRNA subgroup among all these cancer types, while only bladder cancer (BC) and prostate cancer (PC) group with significantly greater diagnostic sensitivity with their multiple miRNA detection. Secondly, none of these cancer types showed significant differences on diagnostic sensitivity and specificity in their specimen and sample size subgroups. Thirdly, the diagnostic sensitivity between Asian (0.791, 95% CI: 0.748–0.827) and Caucasian (0.713, 95% CI: 0.666–0.756) in BC type was shown significant different with the *P*-value of 0.011.

The results of our study suggested that miRNAs, particularly the multiple miRNAs, may play an important role in diagnosis and monitoring of the urologic cancers as superior biomarkers.

## Abstract 2

Supplemental Digital Content is available in the text

## INTRODUCTION

Urologic cancers are abnormal cell growth that occurs in the kidney, bladder, prostate, and testicles. Overall, about 1,607,602 new cases of urologic cancers were diagnosed worldwide in 2008, which ranked the second most common group of human malignancies.^1^ The main etiological factors of urologic cancers are considered to be occupational carcinogen exposure and smoking habit. Renal cell carcinoma (RCC), the most common neoplasm in kidney, accounts for nearly 3% of adult malignancies and its mortality rate is over 40%.^2,3^ RCC can be divided into several subtypes, such as clear cell (ccRCC), chromophobe (chRCC), and papillary (pRCC). The 5-year survival rate of RCC ranges from 15% to 50%, depending on tumor stage, treatment, and selection.^4^ For bladder cancer (BC), most of them are nonmuscle-invasive (NMIBC) lesions. Muscle-invasive (MIBC) lesions account for about 20% of the annual incidence of BC, amounting to approximately 15,000 deaths per year in the United States.^1^ The 5-year survival rate of BC ranges widely in different stages. The patients at early stage (I or II) are more likely to survive with a 5-year survival rate of 97%, while the patients at advanced stage (III or IV) have little chance to survive with only a 5-year survival rate of 6% since the tumor has already spread or invaded to other organs, which make it quite difficult to remove or kill cancer cells.^5^ Furthermore, prostate cancer (PC) is the second most common noncutaneous cancer in males. The potential environmental risk factors of PC were found in previous studies, such as diet. PC, due to its gradual progression and nonspecific symptoms, is usually diagnosed advanced stage. However, if PC patients could be diagnosed at its early stage, the treatment success rate would be greatly improved. Therefore, early detection of urologic cancers is both important and necessary for cancer patients to receive timely treatment.

Currently, the conventional diagnostic methods of urologic cancers consist of urine-based test, cystoscopy, and upper-tract imaging. The urine cytology exhibits a high specificity (around 95%) but a low sensitivity (around 30%) for BC.^6^ The cystoscopy achieves the visualization of the internal urethra and bladder, which facilitates the fast and accurate diagnosis of urological diseases. But it may cause discomfort on patients during the process, and the invasiveness of cystoscopy may affect the life quality of patients. Moreover, the cystoscopy is a relatively expensive clinical examination.^7^ Early cancer detection could help patients manage their cancer in a better way, thus improve the survival rate of patients. The challenge for early cancer detection is that nonspecific symptoms could be observed easily at the early stage of cancer. Therefore, a novel noninvasive diagnostic biomarker with not only high sensitivity and specificity, but also friendly nature to patients, is urgently needed.

MicroRNA (miRNA) is an abundant class of small noncoding RNAs, with 20 to 25 nucleotides in length. MiRNA are reported to be involved in the biological processes related to tumorigenesis, including cell proliferation, differentiation, and apoptosis.^8–10^ Since the first miRNA was discovered, miRNAs had been regarded as transcriptional byproducts for a long time. Not until miRNAs were found to be specifically deleted in leukemia did people realize the importance of them.^11^ Subsequent studies found that miRNAs are highly associated with various human cancers, including urologic cancers. For example, 4 miRNAs (miR-28, -185, -27, and -7f-2) were found significantly upregulated in RCC patients compared with healthy people. Overexpression of 10 miRNAs (miR-223, -26b, -221, -103-1, -185, -23b, -203, -17-5p, -23a, and -205) was observed as well in BC patients.^12^ Besides, miRNA-21 expression distinguished pRCC and ccRCC from chRCC with 83% sensitivity and 90% specificity.^13^ MiRNA-375, -143, and -145 were also found with high level sensitivity and specificity in PC detection. Furthermore, it is found that miRNAs exist in blood, urine, and tissue with stable concentration. Therefore, compared with DNA or protein, miRNA might be a predominant biomarker from the biological standpoint.

Subsequent studies have indicated that unique miRNA may lead to the urologic cancers. However, studies with inconsistent results have also been reported. In order to further explore the clinical applicability of miRNAs for urologic cancers, we conducted this systematic meta-analysis based on all relevant studies.

## MATERIALS AND METHODS

### Ethnic Statement

Ethnic approval is not necessary for this meta-analysis.

### Search Strategy

We conduct this meta-analysis under the diagnostic meta-analysis guidelines.^14^ To retrieve all studies concerning the diagnostic value of miRNA in urologic cancer, a comprehensive literature search in Embase, Sinomed electronic databases, Chinese National Knowledge Infrastructure (CNKI), and the Medline (updated to May 30, 2015) was performed without publication date or language restrictions. The following search terms (“miR∗” or “miRNAs” or “microRNAs”) and (“diagnostic value” or “diagnoses” or “receiver operating characteristics” or “ROC curve” or “sensitivity and specificity”) and (“urologic cancers” or “kidney neoplasms” or “ureteral neoplasms” or “urethral neoplasms” or “urinary bladder neoplasms” or “bladder cancer” or “prostate cancer” or “testical cancer”) were used to retrieve all the relevant articles. In addition, reference list of each relevant study was manually searched to obtain other valuable articles.

### Inclusion and Exclusion Criteria

Studies qualified to be included in this meta-analysis had to fulfill the following criteria: all patients were diagnosed urologic cancers using the diagnostic gold standard; case–control studies were included; concerned the utility of miRNAs expression profiles for urologic cancers diagnosis; and including sufficient data (4 values for false negatives [FN], true negatives [TN], true positives [TP], and false positives [FP]). The exclusion criteria were as follows: reviews, case reports, and meta-analyses; articles not related to the diagnostic value of miRNAs for urologic cancers; and studies without valid data (FN, TN, TP, FP, sensitivity, and specificity).

### Data Extraction and Quality Assessment

The following data and information was extracted by 2 independent reviewers: name of the first author, publication year, country, characteristics of participants (ethnicity, sample size, mean/median age, and male ratio), sample source, methods of miRNAs testing, miRNAs profiled, and the data (FN, TN, TP, FP, sensitivity, and specificity). The revised Quality Assessment of Diagnostic Accuracy Studies (QUADAS-2) criteria were used to evaluate the methodological qualities of selected studies.^15^ This QUADAS-2 tool, containing a 7-item checklist (each of which is scored as yes, unclear or no), is comprised of 4 key domains: index test, reference standard patient selection, and flow and timing. It is specifically developed to assess studies’ applicability and risk of publication bias. An answer of “yes,” with a score of 1, means that the bias has a low risk and a high concern. An answer of “unclear,” given a score of 0.5, means that the bias has a moderate risk and a moderate concern. In addition, a score of 0 was given for answers of “no,” which means the bias has high risk and low concern.

### Statistical Analysis

The specificity [TN/(TN + FP)], sensitivity[TP/(TP + FN)], negative likelihood ratio (NLR) [(1-specificity)/specificity)], positive likelihood ratios (PLR) [(sensitivity/(1-sensitivity)], and diagnostic odds ratio (DOR) with the 95% confidence intervals (95% CIs) were performed using the bivariate random-effects regression model.^16^ The DOR, combining the strengths of sensitivity and specificity, is a measurement of test performance, ranging from 0 to infinity.^17^ Simultaneously, summary receiver operator characteristic (SROC) curve was constructed with the sensitivity and specificity of each included study, and the area under the SROC curve (AUC) was calculated.^18^ The AUC ranges from 0.5 for random performance to 1.0 for perfect discrimination, which can be statistically interpreted as the probability of correctly distinguishing patients from the control. The heterogeneity between studies was evaluated using a *Q* test and the I^2^ statistics. The sources of heterogeneity were investigated using meta-regression and stratified analyses (ethnicity, cancer types, miRNAs profiling, and sample types). The random-effects model was applied if a *P*-value less than 0.05 and the Q test or I^2^ values ≥50%, which indicated substantial heterogeneity.^19^ In addition, based on the characteristics of the included studies, we performed subgroup analyses and meta-regression to explore the sources of heterogeneity between studies. All analyses were performed using R (version 3.2.0) statistical software.

## RESULTS

### Search Results

**FIGURE 1:**
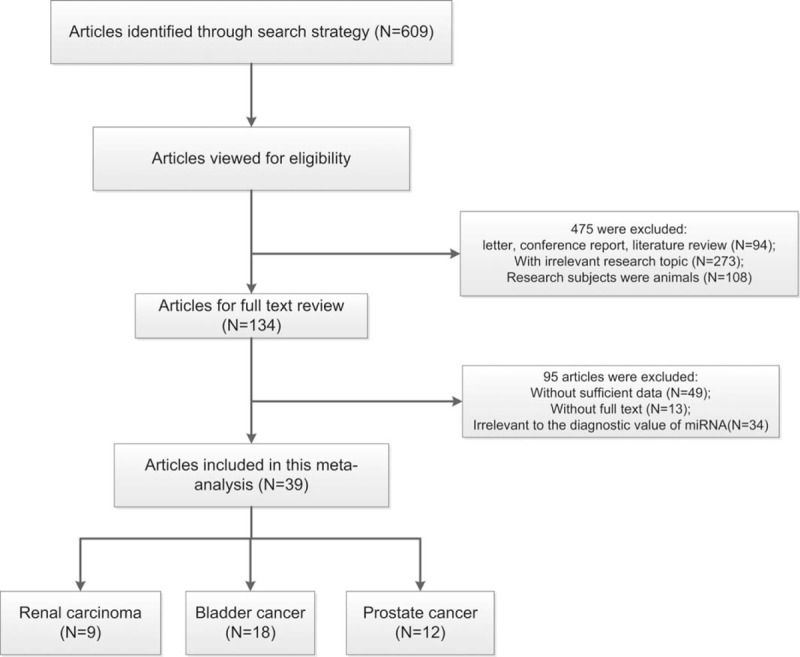
Flowchart of studies selection in this meta-analysis.

Figure 1 presents the results of our literature research. A total of 609 records were first identified by our search strategy without duplicates. After reviewing their abstracts and titles, 475 articles were removed due to unfit literary forms, irrelevant research topic, and subjects of animal model. The rest 134 articles were left for further full-text evaluation. Among these articles, 34 articles are irrelevant to the diagnostic value of miRNAs in urological cancer, 49 articles fail to provide sufficient data for meta-analysis, and 13 articles full texts were not found, with a total of 95 articles were excluded. Finally, 39 articles were included in our meta-analysis, 9 on RCC, 18 on BC, 12 on PC.^6,13,20–41^ Because none of the articles provided sufficient data for diagnostic value of miRNA for testical cancer, our meta-analysis did not include testical cancer.

### Characteristics of Studies

Table 1 , in alphabetical order by surname of first author, summarizes main characteristics of the 39 included articles. All of these selected studies, with the publication years ranging from 2008 to 2015, included a total of 3223 BC, PC, and RCC patients and 2297 controls. Among the 39 articles, 12 articles were conducted in Asian, and the rest 27 articles were conducted in Caucasian. The diagnostic performances of single and multiple miRNA have been investigated among those included articles. As for the specimen types, blood specimens were included in 13 studies, urine in 13 studies, and tissue in 8 articles, 3 articles included 2 specimen types.

**TABLE 1:**
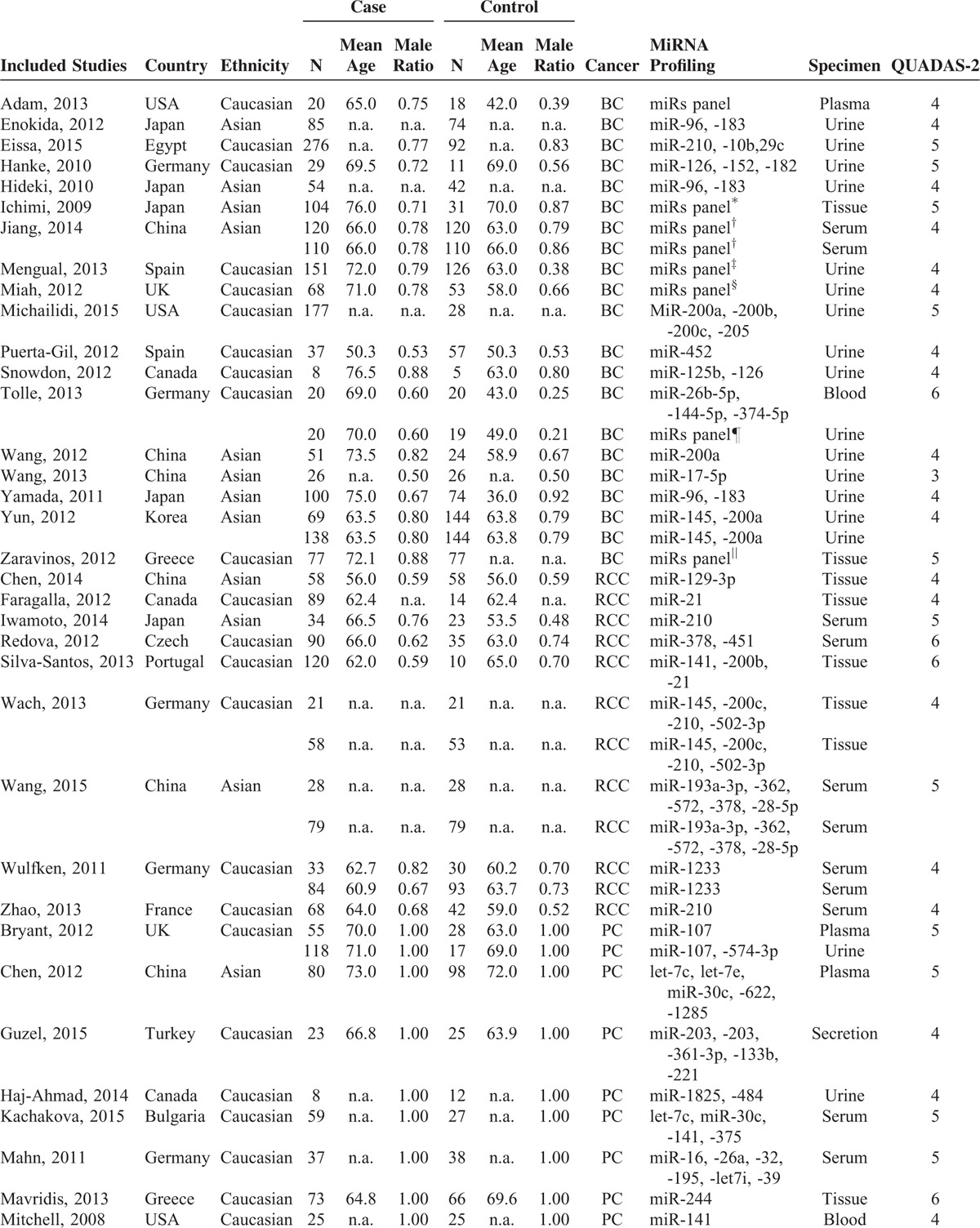
Characteristics and Quality Assessment of Diagnostic Clinical Trials Included in the Meta-Analysis

**TABLE 1 (Continued):**
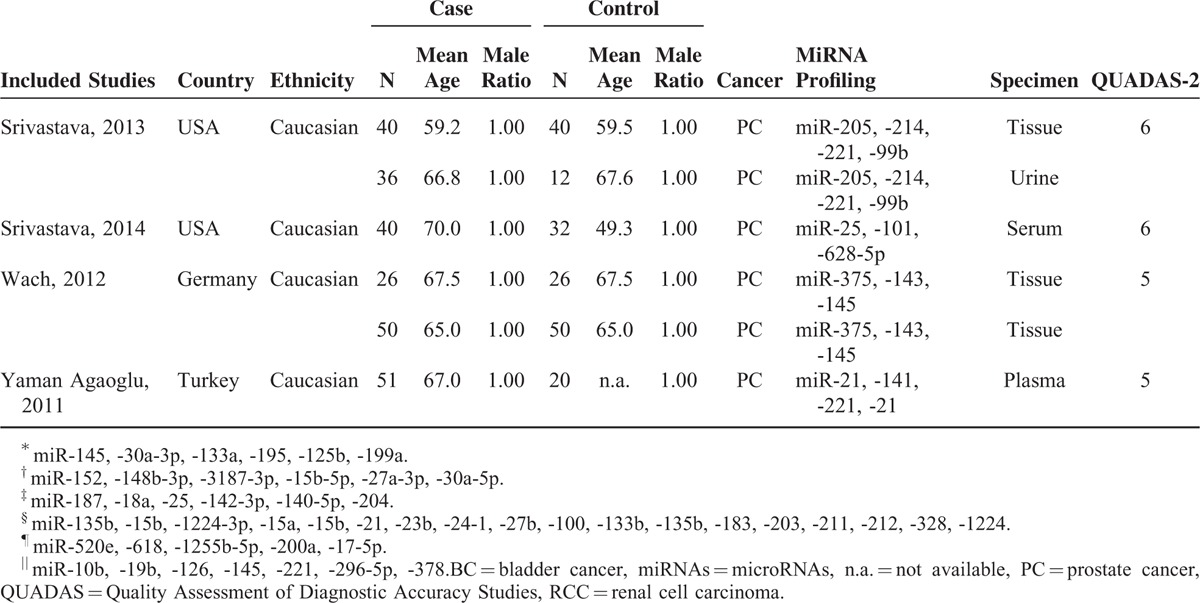
Characteristics and Quality Assessment of Diagnostic Clinical Trials Included in the Meta-Analysis

### Diagnostic Accuracy of RNA Profiling in Urologic Cancers

**FIGURE 2:**
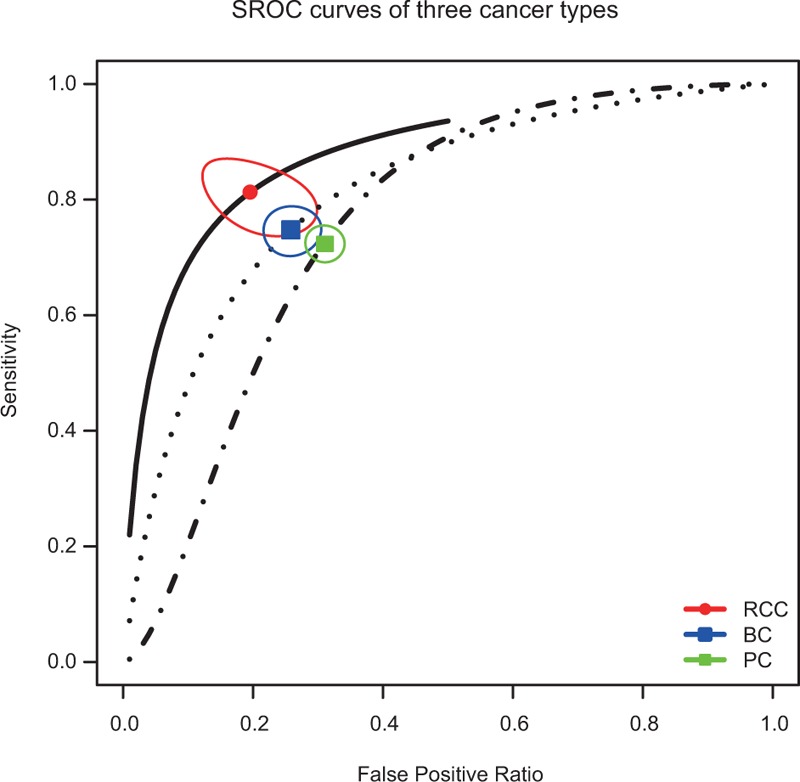
SROC curve of miRNA assay for the diagnosis of urologic cancers.

The diagnostic accuracy of miRNAs in urologic cancers detection is listed in Table 2, pooled from all studies. The heterogeneities between studies of sensitivity and specificity were 85.83% (95% CI: 83.05, 88.62) and 82.63% (95% CI: 79.02, 86.25), respectively. Thus, the random-effects model was used to calculate the pool estimates in this study. Overall, the pooled sensitivity and specificity were 0.744 (95% CI: 0.723–0.763) and 0.724 (95% CI: 0.703–0.744) with an AUC of 0.795. The SROC curve of different cancer types are shown in Figure 2, with AUC values of 0.875 for RCC, 0.808 for BC and 0.755 of PC.

**TABLE 2:**
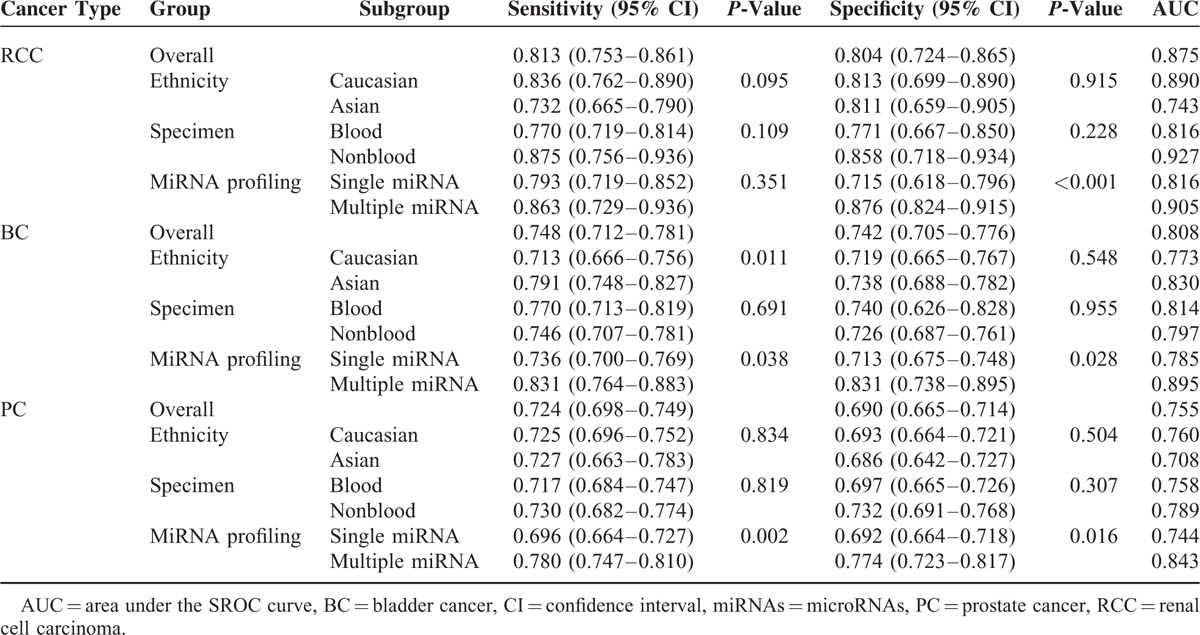
Summary Diagnostic Performance of miRNAs for Urologic Cancers

### Subgroup Analyzing

**FIGURE 3:**
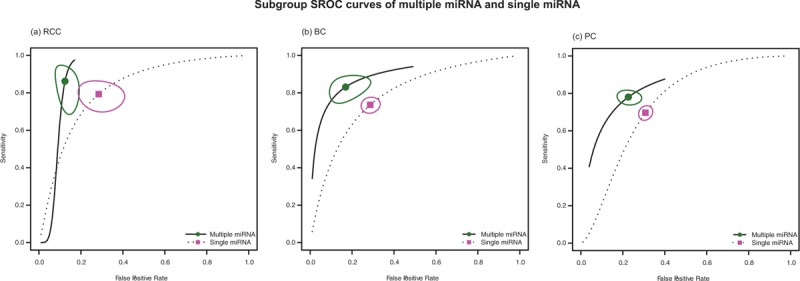
Subgroup SROC curves of multiple or single miRNA assay for the diagnosis of renal cell carcinoma (RCC), bladder cancer (BC), and prostate cancer (PC).

Subgroup analyses based on ethnicity, cancer types, miRNA profiling, and specimen types and sample size were also conducted. The pooled sensitivity, specificity, and AUC for each subgroup are listed in Table 2. As for cancer type, the pooled sensitivity and specificity for RCC were 0.813 (95% CI: 0.753, 0.861) and 0.804 (95% CI: 0.724, 0.865), for BC were 0.784 (95% CI: 0.712, 0.781) and 0.742 (95% CI: 0.705, 0.776), and for PC were 0.724 (95% CI: 0.698, 0.749) and 0.690 (95% CI: 0.665, 0.714), respectively. Figure 2 shows that the diagnostic performance of miRNA was greater in RCC group than in the rest 2 cancer types. Furthermore, for each cancer type, the sensitivity and specificity were also calculated for their ethnicity, specimen, and miRNA profiling subgroups. The details are also presented in Table 2. Each subgroup has a corresponding AUC value. The miRNA profiling subgroup showed significant differences in their diagnostic value within each cancer type (*P*-value of sensitivity difference in BC and PC subgroup were 0.038 and 0.002, respectively; *P*-value of specificity difference in RCC, BC, and PC subgroup were <0.001, 0.028, and 0.016, respectively), except for the *P*-value of RCC sensitivity with 0.351. We found the accuracy of miRNA combination assay significantly higher than that of single miRNA, with their corresponding values presenting in Table 2. Moreover, the SROC curves of each cancer types by miRNA are displayed in Figure 3, which visually shows that the AUC values of multiple miRNA were higher than single miRNA of each cancer type. The specific values of AUC with each subgroup are listed in Table 2. While the ethnicity subgroup analyses showed the only significant difference of their diagnostic performance in the BC group between Asian and Caucasian (*P*-value of sensitivity: 0.011). The specimen and sample size subgroup analyses showed no significant difference in their diagnostic performance (Supplement Figures, http://links.lww.com/MD/A396).

## DISCUSSION

Although great progress occurred in diagnostic techniques, the accurate and convenient diagnosis for urologic cancers still remains clinical challenges. Subsequently, a growing number of studies have reported the potential of miRNAs as accurate biomarkers in urologic cancers diagnosis.^28,32,33^ Since the existing biomarkers do not have a high sensitivity and specificity in urologic cancer detection, the miRNAs which showed high accuracy in urologic cancer diagnosis attract much attention.^26,42^ MiRNAs have more advantages as powerful biomarker in urologic cancer detection than other biomarkers: they are reproducibly and remarkably stable in body fluids, such as blood and urine; the dysregulation of miRNA are closely associated with certain types of cancer; and no invasive procedure is needed. The high stability of miRNAs is possibly due to the combination with other molecules like protein or lipids, which protected themselves from RNase degradation.^43^ Besides, a number of studies imply that miRNAs are specifically correlated with urologic cancers. Wang et al^29^ reported that the downexpression of miRNA-200a was positively correlated with BC risk. Faragalla et al^13^ found that miRNA-21 was significantly upregulated in RCC tissues. Moreover, Wulfken et al^23^ identified an aberrant expression of miR-1233 in cancer patients, compared with healthy controls. Similarly, Chen and his colleagues first identified the different plasma miRNA expression level between PC and controls in China. They have found let-7e, let-7c, and miR-30c were downregulated in PC patients, while miR-622 and miR-1285 were upregulated in PC patients.^44^ The mechanisms that miRNAs involve in urological tumorigenesis could be attributed to the ability of miRNAs, which inhibit translation of oncogenes and tumor suppression. MiRNAs in body fluids may also have functional roles associated with the surrounding tissues. More details and further insight into the mechanisms of carcinogenesis are still urgently needed.

However, due to the different study designs and study subjects, the wide ranges of diagnostic performance were difficult to be compared. Therefore, this study aims to summarize the result of individual studies, and to investigate the diagnostic value of miRNAs for urologic cancers detection. After analyzing and plotting all included data, we found the overall sensitivity and specificity of miRNAs were 0.744 and 0.724 with an AUC value of 0.795.

To explore the potential sources of heterogeneity, subgroup analyses were conducted based on ethnicity, cancer types, specimen types, and sample size. The results of subgroup analysis based on ethnicity suggest that the miRNA expression profile test have no significant difference between Asian and Caucasian group. As for the subgroup analysis of cancer types, we found that RCC assay has a higher accuracy compared with BC and PC assay, the sensitivity, specificity, and AUC of RCC are 0.813, 0.804, and 0.875, which indicated that the miRNAs have the highest diagnostic performance in RCC patient. As for single and multiple RNA, multiple-miRNA shows a superior performance than single-miRNA within each cancer type.

Although we made every effort to limit the bias, there are still several limitations should be noticed in our study. First of all, during our literature search, several valuable studies may be missed although we have already performed a comprehensive search strategy to retrieve all the relevant studies. In addition, some publications in other languages, such as German and French, are not included in this study, which may have influence on publication bias. Furthermore, there were only Asian and Caucasian included in this meta-analysis. Therefore, the association between miRNA expression and urologic cancer in other ethnicities, such as Spanish, African or African-American, might need further research. Secondly, although miRNA is stable in body fluid, the expression level of miRNA might decrease after surgeries. Therefore, the time of sample collection may affect the detection results. Thirdly, the small sample size in our subgroup analysis may lead to the bias in the results. Finally, most of the included publications did not provide the follow-up study, thus made it unable to verify whether controls would be at the risk of developing urological cancers or not later.

In summary, the results of our study shown that miRNAs, particularly the combination usage of miRNAs, have a great potential to be an accurate biomarker to diagnose urologic cancer.
